# Adverse childhood experiences and hypertension: examining the roles of depressive symptoms and cardiometabolic dysregulations based on CHARLS data

**DOI:** 10.3389/fpubh.2025.1567400

**Published:** 2025-06-24

**Authors:** Xuemei Huang, Huiqiong Song, Shuyang Liu, Limeihui Gong, Rui Miao

**Affiliations:** ^1^Department of Humanities and Social Sciences, Zhuhai Campus of Zunyi Medical University, Zunyi, China; ^2^Basic Teaching Department, Zhuhai Campus of Zunyi Medical University, Zunyi, China

**Keywords:** hypertension, adverse childhood experiences, depressive symptoms, cardiometabolic dysregulations, public health

## Abstract

**Objective:**

This study aims to explore the relationship between Adverse Childhood Experiences (ACEs), depressive symptoms, and cardiometabolic dysregulations in the context of hypertension risk, while examining both psychological and physiological factors contributing to the development of hypertension.

**Methods:**

Prospective data were sourced from the China Health and Retirement Longitudinal Study (CHARLS). The data used in this study were collected from the baseline survey in 2011, with follow-up data obtained in 2013 and 2015, covering a 4-year period. Depressive symptoms and cardiometabolic dysregulations were derived from the 2011 baseline survey. Data on ACEs were obtained from the 2014 survey. Incident hypertension cases were defined as new cases of hypertension during the follow-up period (2013 and 2015) among participants who were not hypertensive in 2011. A univariate logistic regression analysis was conducted for correlation analysis, and a path analysis using structural equation modeling (SEM) was performed. The total baseline sample consisted of 6391 participants, with 434 new cases of hypertension during the follow-up period.

**Results:**

The study found that 50.23% of the population had experienced at least one ACE. ACEs were associated with an increased risk of hypertension, Each additional ACE raised the odds of developing hypertension based on systolic blood pressure by 25.2% (OR 1.252 [95% CI 1.103, 1.648], *p* = 0.014). No significant association was observed with diastolic blood pressure. The SEM indicated that ACEs did not have a direct effect on hypertension. Instead, the relationship was mediated by depressive symptoms and cardiometabolic dysregulations. The indirect effect along the ACEs → cardiometabolic dysregulations → SBP pathway was 0.066 (95% CI 0.002, 0.105, *p* = 0.002), and for the ACEs → cardiometabolic dysregulations → DBP pathway, the coefficient was 0.013 (95% CI 0.003, 0.124, *p* = 0.002).The study found no evidence supporting depressive symptoms as a significant mediator.

**Conclusion:**

ACEs show a positive association with hypertension occurrence. They may contribute to hypertension development through their influence on cardiometabolic dysregulations.

## 1 Introduction

Adverse Childhood Experiences (ACEs) refer to traumatic events occurring before the age of 18, encompassing a range of experiences such as physical and emotional abuse, neglect, domestic violence, and parental substance abuse or mental illness (1, 2). These adverse events are associated with significant and lasting effects on both mental and physical health. ACEs are retrospectively reported by approximately half of adults in the United States, United Kingdom, and Canada, with research indicating that an increasing number of ACE categories reported correlates with a higher likelihood of chronic diseases and depression in adulthood (3). Notably, individuals with seven or more ACEs have nearly twice the odds of reporting poor self-rated health compared to those with few or no ACEs (4).

Hypertension, a key risk factor for cardiovascular diseases, is prevalent globally, with an estimated 33% of individuals aged 30-79 affected, according to the World Health Organization’s Global Hypertension Report. Recent studies have increasingly underscored the association between ACEs and the development of hypertension in later life. For example, a longitudinal study in Georgia spanning 23 years found that individuals exposed to multiple ACEs had a significantly higher prevalence of hypertension during youth compared to those without such experiences (5). Similarly, research conducted in Riyadh, Saudi Arabia, reported a more than twofold increase in the risk of hypertension in adults who had experienced four or more ACEs compared to those without ACEs (6). Further, data from the Behavioral Risk Factor Surveillance System surveys conducted in 2009 and 2011 highlighted a robust relationship between even a single ACE and the subsequent development of hypertension, dyslipidemia, and diabetes (7). A meta-analysis also reinforced the significant adverse impact of ACEs on adult health, emphasizing the role of dysfunctional family dynamics and negative family environments in the onset and progression of hypertension (8).

ACEs also have profound effects on mental health, particularly with respect to the development of depression. A positive correlation has been consistently observed between the number of ACEs experienced and the risk of depressive symptoms in adulthood. Specifically, individuals exposed to a higher number of ACEs are at greater risk of developing depression (9, 10). A study focusing on university students revealed that those with multiple ACEs reported significantly higher rates of depressive symptoms compared to those who had not experienced such events (11). The impact of ACEs on mental health is not solely direct; rather, it also operates through indirect pathways, such as impaired emotional regulation and lack of social support. Difficulties in emotional regulation, often stemming from ACEs, are recognized as strong predictors of depression in adulthood (12, 13).

Together, these findings highlight the complex and multifaceted ways in which ACEs contribute to both mental health disorders and physical health conditions, such as hypertension, in adulthood. The understanding of these interrelationships is crucial for public health strategies aiming to mitigate the long-term effects of early life adversities on cardiovascular and mental health.

Indirect evidence suggests that depressive symptoms in adulthood may mediate the relationship between ACEs and the development of hypertension. A significant comorbidity exists between depression and hypertension, with multiple studies indicating that individuals with hypertension exhibit a markedly higher prevalence of depression compared to the general population (14–16). This association is bidirectional, where hypertension can exacerbate depressive symptoms, while depression may contribute to the development of hypertension (17, 18). Depression has been identified as a key risk factor for hypertension, with proposed mechanisms involving both endocrine and neurobiological changes. Additionally, depression influences lifestyle behaviors such as poor dietary choices and reduced physical activity, which can further aggravate hypertensive conditions (19, 20). Moreover, sleep disturbances commonly associated with depression have been shown to play a role in the onset of hypertension, as poor sleep quality is a known contributor to elevated blood pressure levels (21).

Furthermore, it is posited that ACEs may influence the risk of hypertension in adulthood through cardiometabolic dysregulations (22). Cardiometabolic dysregulation refers to a cluster of interrelated metabolic and cardiovascular abnormalities, including hypertension, hyperlipidemia, hyperglycemia, systemic inflammation, and obesity, among others. These conditions are known to impact the development of hypertension through various biological pathways (23, 24). Therefore, cardiometabolic dysregulation may represent an alternative pathway linking ACEs to hypertension in adulthood.

In summary, there is a clear association between ACEs and the risk of hypertension in later life. However, the specific mechanisms underlying this relationship remain unclear. We hypothesize that this association is not the result of a direct effect of ACEs on hypertension, but rather through a complex interplay of psychological and biological pathways that indirectly contribute to the condition. Existing research has demonstrated that ACEs are associated with the development of depressive symptoms in adulthood and with cardiometabolic dysregulation, both of which are recognized as significant risk factors for hypertension. Therefore, we propose that depressive symptoms and cardiometabolic dysregulation may serve as crucial mediators linking ACEs to the heightened risk of hypertension in later life. However, to our knowledge, no previous studies have simultaneously examined these potential mediators in a longitudinal pathway model. This study is guided by the life course perspective, which emphasizes that early life events influence health outcomes in middle and later life through cumulative biological, psychological, and social processes (25). Accordingly, the proposed model examines the pathway from ACE to hypertension onset, mediated by midlife factors such as depressive symptoms and cardiometabolic dysregulations. In this framework, ACEs are conceptualized as critical turning points in early life. The study adopts a longitudinal design, tracking participants from 2011 to 2015 to capture the progression of health changes over time. Both psychological (depressive symptoms) and physiological (metabolic dysregulations) mediators are included, aligning with the life course theory’s concept of multilayered mediation pathways (26).

The aim of this study is to investigate the potential mediating role of depressive symptoms in the relationship between ACEs and hypertension using data from the CHARLS. Previous research utilizing CHARLS data has demonstrated that individuals with four or more ACEs face a significantly increased risk of mental health disorders, including a 265% rise in the prevalence of depression (27, 28). Additionally, findings suggest that a greater accumulation of ACEs is associated with heightened risks of depressive symptoms and cognitive decline (28, 29). The association between ACEs and various chronic diseases, such as cardiovascular disease, diabetes, and oral health issues, highlights the long-term consequences of early adverse experiences on the risk of developing multiple chronic conditions in adulthood (30–32). Despite these established links between ACEs and various health outcomes, the specific relationship between ACEs and hypertension, and the potential mediating factors involved, remain underexplored. Thus, this study seeks to empirically test the hypothesis that depressive symptoms act as an independent mediator in the association between ACEs and adult hypertension, thereby enhancing our understanding of how early life adversities can influence cardiovascular health later in life.

In summary, this study offers innovation in three key areas. First, it focuses specifically on middle-aged and older Chinese adults by using the nationally representative CHARLS cohort, addressing the limited evidence on the ACEs-hypertension link in non-Western populations. Second, it advances the field by examining depressive symptoms and cardiometabolic dysregulations simultaneously as mediators within a unified analytical model. This integrated approach contrasts with prior research that typically investigates these pathways separately. Third, the study demonstrates methodological rigor through its prospective design, identifying incident hypertension cases to support temporal inference. It models mediators as latent variables within structural equation modeling to reduce measurement error. Additionally, it quantifies cumulative ACE exposure using a continuous score and clearly distinguishes new hypertension cases among participants who were normotensive at baseline.

## 2 Research methods and design

### 2.1 Study participants

This study utilized data from the China Health and Retirement Longitudinal Study (CHARLS), a nationally representative longitudinal survey initiated in 2011. The survey collects micro-level data on Chinese individuals aged 45 and older. The baseline survey, conducted in 2011, included over 10,000 households across 150 counties and 450 villages randomly selected from across the country. The primary objective of CHARLS was to examine various aspects of health, economic status, and social conditions among older adults. Subsequent follow-up surveys were conducted in 2013, 2015, 2018, 2020, and 2021-2023. Additionally, the “Life History Survey” was conducted in 2014 (33). The CHARLS survey protocol was approved by the Biomedical Ethics Committee of Peking University (Approval No: IRB00001052-11015). Written informed consent was obtained from all participants prior to their involvement in the study.

### 2.2 Measures

#### 2.2.1 Assessment of ACEs

ACEs refer to potentially traumatic events individuals may have encountered before the age of 18. In this study, ACEs were assessed using a modified version of the ACEs scale developed by the Centers for Disease Control and Prevention (34), incorporating relevant literature (35, 36) and CHARLS data. The ACEs scale included nine categories of adverse experiences:

Physical Abuse: Whether a female or male guardian ever physically hit the participant (often or sometimes).

Emotional Abuse: The quality of the relationship with the guardian, rated as poor.

Physical Neglect: Instances in which the female guardian failed to provide adequate care.

Emotional Neglect: Situations in which a male or female guardian exhibited favoritism toward a sibling or demonstrated severe gender bias.

Divorce of Biological Parents: Whether the participant’s biological parents were ever divorced.

Mental Illness in Guardians: Whether a guardian suffered from depression lasting at least 2 weeks or exhibited mental health abnormalities.

Witnessing Domestic Violence: The frequency of parental arguments or physical altercations observed by the participant.

Substance Abuse: Whether a guardian struggled with alcoholism, drug use (including opiate use).

Incarceration of Family Members: Whether a guardian was ever sentenced to prison.

Each ACE was coded as 1 for the presence of the experience and 0 for its absence, resulting in a composite ACE score ranging from 0 to 9. This scoring system allowed for quantification of ACE exposure levels among participants and facilitated the investigation of their potential impact on hypertension and associated mediating factors.

#### 2.2.2 Assessment of hypertension

Hypertension was assessed based on the criteria for primary hypertension, defined as a systolic blood pressure (SBP) ≥ 140 mmHg and/or a diastolic blood pressure (DBP) ≥ 90 mmHg (37). In addition to objective blood pressure measurements, participants were asked whether they had ever been diagnosed with hypertension, with those answering “yes” categorized as self-reported hypertensive individuals. Participants who reported taking antihypertensive medication were also included in the hypertensive group. If participants self-reported a diagnosis of hypertension but their measured blood pressure fell within the normal range, the classification was based on the measured values. Blood pressure measurements were taken three times using an oscillometric sphygmomanometer, and the average of the three readings was used to ensure accuracy and reliability in the assessment of hypertension.

#### 2.2.3 Assessment of depressive symptoms

Depressive symptoms in the CHARLS dataset were assessed using the 10-item Center for Epidemiologic Studies Depression Scale (CES-D10) (38). This abbreviated version of the scale measures the frequency of depressive symptoms experienced by participants over the past week. It comprises 10 items spanning three domains: five items related to somatic symptoms, three items addressing depressive mood, and two items reflecting positive affect. Each item is rated on a 4-point scale (ranging from 1 to 4), corresponding to the frequency of symptom occurrence. The total score ranges from 10 to 40, with a CES-D10 score of ≥20 indicative of clinically significant depressive symptoms and a score <20 suggesting the absence of such symptoms. Higher scores correspond to more severe depressive symptoms. The CES-D10 has demonstrated strong internal consistency, with a Cronbach’s alpha coefficient of 0.891 (38), supporting the scale’s reliability.

#### 2.2.4 Assessment of cardiometabolic dysregulations

Cardiometabolic dysregulations were assessed following established criteria outlined in previous research (39, 40). Key indicators of dysregulations included hyperlipidemia, hyperglycemia, systemic inflammation, and obesity. Hyperlipidemia was defined by triglyceride levels >200 mg/dl and HDL cholesterol levels <40 mg/dl. Hyperglycemia was determined through fasting blood glucose levels ≥126 mg/dl. Systemic inflammation was measured using C-reactive protein (CRP) levels ≥3 mg/L, and obesity was assessed by waist circumference, with central obesity defined as a waist circumference ≥90 cm for men and ≥85 cm for women. These measurements adhered to standardized protocols to ensure their reliability and validity (33, 41).

### 2.3 Study design

**Figure 1:**
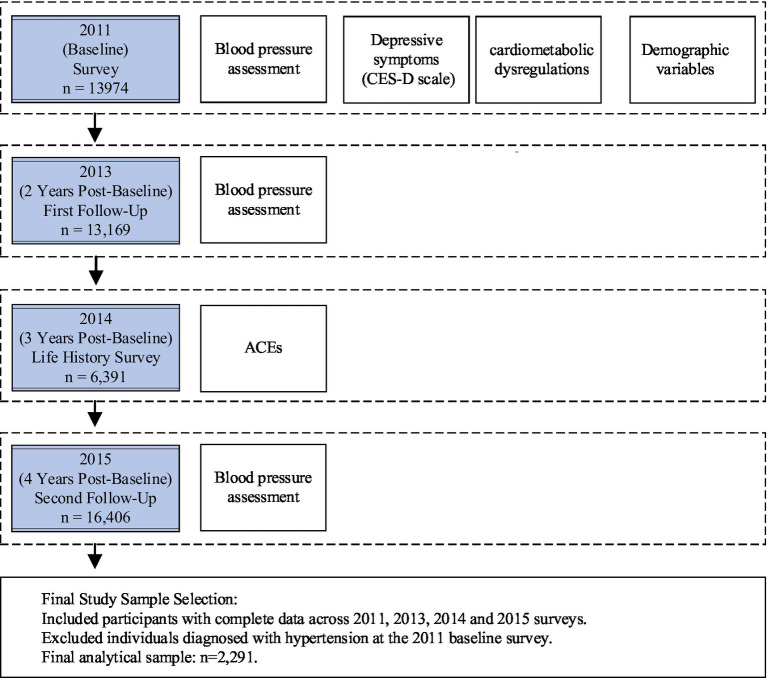
CHARLS data sample collection and processing workflow.

The study timeline and data collection points are presented in Figure 1. This research adopted a prospective design, utilizing data from the CHARLS, which began with a baseline survey in 2011 and included follow-up assessments through 2013 and 2015, spanning a total of 4 years. Data on depressive symptoms and cardiometabolic dysregulations were obtained from the 2011 baseline survey. Information on ACEs was derived from the “CHARLS Life History Data” collected in 2014, as the baseline survey did not include ACEs data. Participants were asked to retrospectively recall childhood experiences, minimizing potential bias in the analysis. Missing demographic data were supplemented by matching with the 2011 dataset.

Hypertension data were derived from the follow-up assessments, identifying individuals who developed hypertension during the 2013 and 2015 follow-ups. Participants with pre-existing hypertension reported in the 2011 baseline were excluded from the analysis. The complete dataset for ACEs from the 2014 survey included *N* = 16,133 respondents. After excluding those with incomplete data for CES-D10, cardiometabolic dysregulations, and hypertension measures, the final sample included *n* = 6,391 participants, with 434 new cases of hypertension during the follow-up period.

### 2.4 Statistical analysis

Statistical analyses were performed using SPSS 24.0 and MPlus 8.7. Descriptive statistics were used to summarize the characteristics of the study participants, with frequencies and means reported for categorical and continuous variables, respectively. To explore the relationships between ACEs, depressive symptoms, cardiometabolic dysregulations, and hypertension, univariate binary logistic regression analysis was conducted.

To investigate potential mediation effects, structural equation modeling (SEM) was employed. In this model, ACEs were treated as a continuous independent variable, with depressive symptoms and cardiometabolic dysregulations acting as mediating variables. Depressive symptoms were conceptualized as a latent variable, measured by the 10 items of the CES-D10 scale, while cardiometabolic dysregulations were treated as another latent variable, incorporating observable indicators such as triglycerides, HDL cholesterol, fasting blood glucose, CRP, and waist circumference. Initial analyses involved estimating path models for each of the two mediating variables independently. Subsequently, the mediation effects between depressive symptoms and cardiometabolic dysregulations were examined.

### 2.5 Common method bias test

As part of the data in this study was collected through self-report surveys, Harman’s single-factor test was employed to assess the potential presence of common method bias. Exploratory factor analysis revealed that the variance explained by the largest common factor was 18.73%, which is substantially below the 40% threshold typically used to indicate significant common method bias. These results suggest that common method bias is unlikely to pose a substantial concern in this study.

## 3 Results

### 3.1 Participant demographics

#### 3.1.1 Analysis of ACEs prevalence

As shown in Table 1, a total of 6,391 participants were included in the baseline analysis, with 3,014 identified as male. The gender distribution did not exhibit a statistically significant difference. The majority of participants were married (98.6%), with a substantial proportion residing in rural areas (79.1%). Educational attainment was predominantly at or below the high school level, representing 96.1% of the sample. Regarding ACEs, 3,210 participants (50.23%) reported having experienced at least one type of ACE from the nine categories assessed. The most common category was physical abuse, which affected 28.3% of participants. Mental health issues of guardians ranked second in prevalence. The category with the lowest occurrence was guardian criminal behavior, reported by only 0.3% of participants (see Table 1).

**Table 1:** Percentage of baseline participants’ reported statistics by ACEs category and selected features.

Characteristic		Survey population *n* (%)	Physical abuse *n* (%)	Physical neglect *n* (%)	Emotional abuse *n* (%)	Emotional neglect *n* (%)	Biological parents divorced *n* (%)	Mentally ill guardian *n* (%)	Substance abusing guardian n (%)	Guardian in prison *n* (%)	Witness domestic violence *n* (%)
Total		6,391	1808 (28.3)	108 (1.7)	392 (6.1)	418 (6.5)	33 (0.5)	1,547 (24.2)	48 (0.8)	22 (0.3)	684 (10.7)
Gender	Male	3,041 (47.6)	1,039 (34.17)	50 (1.64)	122 (4.01)	181 (5.95)	15 (0.49)	709 (23.31)	25 (0.74)	14 (0.46)	304 (10.00)
	Female	3,350 (52.4)	769 (22.96)	58 (1.73)	270 (8.06)	237 (7.07)	18 (0.54)	838 (25.01)	23 (0.69)	8 (0.24)	380 (11.34)
Marriage	Yes	6,304 (98.6)	1783 (28.28)	105 (1.67)	389 (6.17)	414 (6.57)	32 (0.51)	1,522 (24.14)	46 (0.73)	21 (0.33)	678 (10.76)
	No	87 (1.4)	25 (28.74)	3 (3.45)	3 (3.45)	4 (4.60)	1 (1.15)	25 (28.74)	2 (2.30)	1 (1.15)	6 (6.90)
Birthplace	Rural	5,057 (79.1)	1,444 (28.55)	92 (1.82)	292 (5.77)	314 (6.21)	20 (0.40)	1,330 (26.30)	40 (0.79)	14 (0.28)	550 (10.88)
	Urban	1,334 (20.9)	364 (27.29)	16 (1.20)	100 (7.50)	104 (7.80)	13 (0.97)	217 (16.27)	8 (0.60)	8 (0.60)	134 (10.04)
Age	45-64	2,238 (35.0)	561 (25.07)	47 (2.10)	142 (6.34)	170 (7.60)	12 (0.54)	568 (25.38)	20 (0.89)	13 (0.58)	224 (10.01)
	>64	4,153 (65.0)	1,247 (30.03)	61 (1.47)	250 (6.02)	248 (5.97)	21 (0.51)	979 (23.57)	28 (0.67)	9 (0.22)	460 (11.08)
Education Level	≤High school	6,377 (99.8)	1807 (28.34)	108 (1.69)	390 (6.12)	418 (6.55)	32 (0.50)	1,544 (24.21)	45 (0.73)	22 (0.34)	665 (10.56)
	>High school	14 (0.2)	1 (7.14)	0 (0.00)	2 (14.29)	0 (0.00)	1 (7.14)	3 (21.43)	3 (21.43)	0 (0.00)	0 (0.00)

#### 3.1.2 Participant selection and demographic characteristics

In 2011, 13,974 individuals were surveyed, with 4,969 identified as hypertensive, accounting for 35.56%. The 2013 survey included 13,169 participants, with 4,969 hypertensive cases (38.22%). In 2015, 16,406 individuals were surveyed, among whom 4,969 had hypertension (33.81%). After excluding those with hypertension at baseline in 2011 and matching other variables, 2,291 participants remained for analysis. As shown in Table 2, males comprised 49.02% and married individuals 99.21%. Gender appeared to influence blood pressure. The mean SBP was 138.62 ± 20.09 mmHg in males and 137.22 ± 21.62 mmHg in females, with no significant difference. However, males showed a significantly higher mean DBP of 81.02 ± 13.23 mmHg compared to females at 79.03 ± 11.95 mmHg. This suggests gender may affect DBP. Age also showed an association with blood pressure. Compared to participants aged 65 and older, those aged 45–64 exhibited lower SBP but higher DBP.

**Table 2:** Analysis of participants’ demographic data (*n* = 2,291).

Demographic variables		*n* (%)	SBP (M ± SD)	T	DBP (M ± SD)	T
Gender	Male	1,123 (49.02)	138.62 ± 20.09	1.611	81.02 ± 13.23	3.777***
	Female	1,168 (50.98)	137.22 ± 21.62		79.03 ± 11.95	
Marriage	Yes	2,273 (99.21)	137.82 ± 20.87	−2.242*	79.96 ± 12.63	−1.855
	No	18 (0.79)	148.89 ± 18.51		85.50 ± 11.079	
Birthplace	Rural	1971 (86.03)	137.71 ± 20.88	−1.089	79.94 ± 12.73	−0.571
	Urban	320 (13.97)	139.08 ± 20.95		80.38 ± 11.97	
Age	45-64	1,330 (58.05)	133.49 ± 18.81	−12.297***	80.75 ± 12.79	3.327**
	≥65	961 (41.95)	144.02 ± 22.06		78.97 ± 12.34	
Education level	≤High school	2,285 (99.74)	137.92 ± 20.88	0.732	80.02 ± 12.64	1.134
	>High school	6 (0.26)	131.67 ± 25.98		74.17 ± 5.85	

***p < 0.001;**p < 0.01; *p < 0.05. SBP, Systolic Blood Pressure; DBP, Diastolic Blood Pressure.

During the follow-up periods in 2013 and 2015, an additional 434 participants were diagnosed with hypertension, of which 231 (53.2%) were male. The average depressive symptom score was 20.33 ± 4.81, ranging from 10 to 36. Of those assessed, 340 individuals (78.3%) exhibited at least one abnormal cardiometabolic factor, including elevated triglycerides (15.3%), low HDL cholesterol (3.92%), high fasting blood glucose (35.71%), elevated CRP (19.82%), and increased waist circumference (43.55%).

### 3.2 Correlational analysis

The associations among ACEs, depressive symptoms, cardiometabolic dysregulations, and hypertension were examined using univariate logistic regression. For analytical clarity, blood pressure was categorized into Systolic blood pressure (SBP) and Systolic blood pressure (DBP). The results indicated that ACEs were significantly associated with an increased risk of hypertension during the follow-up period. For each additional ACE experienced, the odds of developing hypertension based on systolic blood pressure increased by 25.2% (OR 1.252 [95% CI 1.103, 1.648], *p* = 0.014). No significant association was found with diastolic blood pressure. Additionally, ACEs were positively correlated with the severity of depressive symptoms (OR 1.540 [95% CI 1.303, 1.821], *p* = 0.000), indicating a significant difference. While ACEs also showed a positive association with cardiometabolic dysregulations, this result did not reach statistical significance (OR 0.855 [95% CI 0.716, 1.102], *p* = 0.087).

Further analysis showed that the presence of cardiometabolic dysregulations was associated with a 56.2% increased risk of elevated SBP (OR = 1.562, 95% CI [1.302, 1.874], *p* < 0.001) and a 49.2% increased risk of elevated DBP (OR = 1.492, 95% CI [1.167, 1.908], *p* = 0.001). These findings suggest a close association between higher cardiometabolic dysregulations and the occurrence of hypertension. Meanwhile, depression showed no significant association with SBP (OR = 0.999, 95% CI [0.845, 1.182], *p* = 1.000) or DBP (OR = 0.906, 95% CI [0.728, 1.127], *p* = 1.000). Moreover, no significant relationship was observed between depression and cardiometabolic dysregulations (OR = 0.998, 95% CI [0.835, 1.193], *p* = 1.000). Therefore, in the subsequent model, depression and cardiometabolic dysregulations were included as parallel mediators.

### 3.3 The mediating role of depressive symptoms and cardiometabolic dysregulations between ACEs and hypertension

#### 3.3.1 Measurement model evaluation

To examine the mediating effects of depressive symptoms and cardiometabolic dysregulations in the relationship between ACEs and hypertension, SEM was employed. Prior to testing the mediation effects, it was essential to validate the measurement model. This model comprised two latent variables: depressive symptoms, assessed via the CES-D, which includes 10 items, and cardiometabolic dysregulations, defined by five observable indicators: triglycerides, HDL cholesterol, fasting blood glucose, C-reactive protein, and waist circumference.

A Confirmatory Factor Analysis (CFA) was conducted under the assumption that depressive symptoms and cardiometabolic dysregulations were uncorrelated, using an orthogonal model with the covariance between the two latent variables set to zero. The model’s fit indices suggested a satisfactory fit: χ^2^/df = 2.97, CFI = 0.917, TLI = 0.905, RMSEA = 0.073. These results support the acceptability of the measurement model and provide a solid foundation for subsequent analysis of the structural model.

#### 3.3.2 Mediating role of depressive symptoms and cardiometabolic dysregulations between ACEs and systolic blood pressure (SBP)

The mediating roles of depressive symptoms and cardiometabolic dysregulations in the relationship between ACEs and SBP were systematically examined. First, a model with depressive symptoms as the sole mediator was tested. This model showed acceptable fit indices: CFI = 0.913, TLI = 0.892, χ^2^/df = 6.01, RMSEA = 0.063, and SRMR = 0.038. Using the bias-corrected bootstrap method with 5,000 resamples to assess mediation, results indicated a positive association between ACEs and depressive symptoms (path coefficient = 0.115, 95% CI [0.018, 0.364], *p* < 0.001). However, depressive symptoms were not significantly related to SBP (path coefficient = −0.121, 95% CI [−0.283, 0.021]). The direct effect of ACEs on SBP was not significant (path coefficient = −0.485, 95% CI [−0.485, 1.343]).

Next, a model with cardiometabolic dysregulations as the sole mediator was evaluated. This model also demonstrated acceptable fit: CFI = 0.828, TLI = 0.747, χ^2^/df = 6.23, RMSEA = 0.065, SRMR = 0.041. The results showed a significant association between cardiometabolic dysregulations and SBP (path coefficient = 0.240, 95% CI [0.134, 0.476], *p* = 0.001). In contrast, the relationship between ACEs and SBP was not significant, with a direct effect confidence interval including zero (95% CI [−0.034, 0.052]).

**Figure 2:**
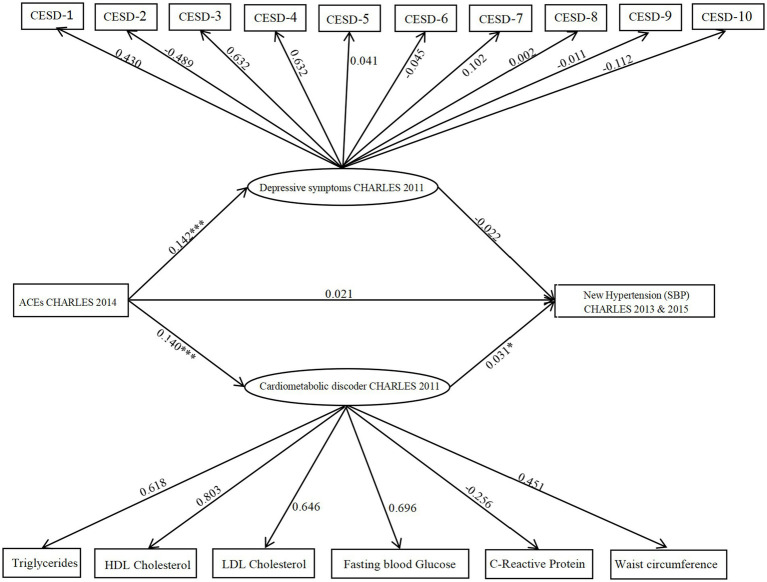
Model illustrating CES-D and cardiometabolic dysregulations as parallel mediators in the relationship between ACEs and hypertension (SBP). The figure displays unstandardized probability regression coefficients. Circles represent latent variables, and rectangles represent observed variables. *p < 0.05; **p < 0.01,***p < 0.001.

Finally, a parallel mediation model including both depressive symptoms and cardiometabolic dysregulations was analyzed. As shown in Figure 2, the combined model fit was satisfactory: CFI = 0.897, TLI = 0.881, χ^2^/df = 2.97, RMSEA = 0.048, and SRMR = 0.037. The results revealed a significant association between ACEs and depressive symptoms (path coefficient = 0.142, 95% CI [0.001, 0.301], *p* = 0.000). The analysis revealed no significant association between depressive symptoms with increased SBP {path coefficient = −0.022, 95% CI [−0.105, 0.117], P (*p* > 0.05)}, the indirect path from ACEs through depressive symptoms to SBP was not significant. Additionally, ACEs were positively associated with cardiometabolic dysregulations (path coefficient = 0.140, 95% CI [0.002, 0.323], *p* = 0.000), which in turn were significantly linked to increased SBP risk (path coefficient = 0.031, 95% CI [0.06, 0.183], *p* = 0.036). The indirect effect via cardiometabolic dysregulations was statistically significant (indirect effect = 0.066, 95% CI [0.002, 0.105], *p* = 0.022). This indicates a significant mediation pathway from ACEs through cardiometabolic dysregulations to SBP.

#### 3.3.3 Mediating role of depressive symptoms and cardiometabolic dysregulations between ACEs and DBP

Following the same steps as above, first, a model with depressive symptoms as the sole mediator was evaluated. The model demonstrated acceptable fit indices: CFI = 0.914, TLI = 0.893, χ^2^/df = 6.12, RMSEA = 0.063, and SRMR = 0.038. Path analysis showed a significant positive association between ACEs and depressive symptoms. Using the bias-corrected bootstrap method with 5,000 resamples to assess mediation, results indicated that ACEs were positively associated with depressive symptoms (path coefficient = 0.096, 95% CI [0.018, 0.364], *p* < 0.001). However, depressive symptoms were not significantly associated with DBP (path coefficient = −0.043, 95% CI [−0.203, 0.198], *p* = 0.087). The direct effect of ACEs on DBP was not significant, with a 95% confidence interval spanning zero (95% CI [−0.031, 0.055]).

Next, a model with cardiometabolic dysregulations as the sole mediator was constructed. This model was acceptable, showing good fit indices: CFI = 0.862, TLI = 0.797, χ^2^/df = 7.98, RMSEA = 0.058, and SRMR = 0.035. Results revealed a significant association between cardiometabolic dysregulations and DBP (path coefficient = 0.110, 95% CI [0.034, 0.476], p = 0.001).

**Figure 3:**
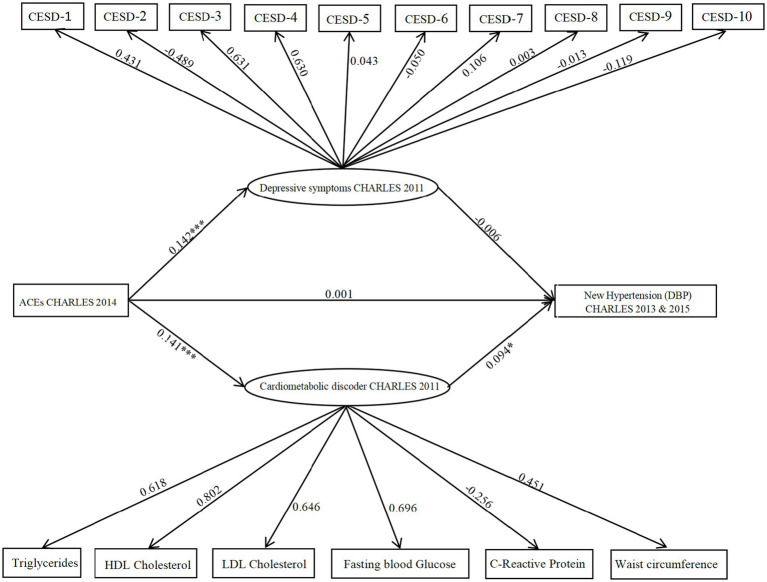
Model illustrating CES-D and cardiometabolic dysregulations as parallel mediators in the relationship between ACEs and hypertension (DBP). The figure displays unstandardized probability regression coefficients. Circles represent latent variables, and rectangles represent observed variables. *p < 0.05; **p < 0.01,***p < 0.001.

Finally, a parallel mediation model including both depressive symptoms and cardiometabolic dysregulations was analyzed (as shown in Figure 3). The combined model showed satisfactory fit indices: CFI = 0.904, TLI = 0.889, χ^2^/df = 2.93, RMSEA = 0.046, and SRMR = 0.035. Findings showed a significant positive association between ACEs and depressive symptoms (path coefficient = 0.142, 95% CI [0.011, 0.321], *p* = 0.001). However, depressive symptoms did not have a significant association with DBP (path coefficient = −0.006, 95% CI [−0.101, 0.214], *p* > 0.05), indicating that the indirect path from ACEs through depressive symptoms to DBP was not significant. Additionally, ACEs were positively linked to cardiometabolic dysregulations (path coefficient = 0.141, 95% CI [0.001, 0.316], *p* < 0.001), which were in turn significantly associated with increased DBP risk (path coefficient = 0.094, 95% CI [0.001, 0.323], *p* = 0.044). The indirect effect of cardiometabolic dysregulations on DBP was statistically significant (indirect effect = 0.013, 95% CI [0.003, 0.124], *p* = 0.002), supporting a significant mediation pathway from ACEs via cardiometabolic dysregulations to DBP.

## 4 Discussion

This study investigates the impact of ACEs on the development of hypertension, focusing on the mediating roles of depressive symptoms and cardiometabolic dysregulations. This study represents a pioneering effort to utilize data from a large prospective cohort to explore both psychological and biological pathways that may connect ACEs to hypertension. Our findings indicate that individuals with a history of ACEs are at a higher risk for developing hypertension, with cardiometabolic disorders playing central mediating roles. These findings collectively suggest that chronic stress stemming from ACEs may trigger hyperactivity of the HPA axis and sustained inflammatory responses, thereby accelerating vascular endothelial dysfunction and autonomic nervous system imbalance. Notably, cardiometabolic dysregulations—such as insulin resistance and dyslipidemia—may mediate and further amplify this cascade via oxidative stress pathways. Future research should validate these mechanisms through longitudinal biomarker assessments, including cortisol and C-reactive protein, and investigate targeted interventions for depressive symptoms to reduce hypertension risk in populations exposed to ACEs.

While our study confirmed cardiometabolic dysregulation as a significant mediator between ACEs and hypertension (particularly for systolic BP), the hypothesized mediating role of depressive symptoms did not reach statistical significance. This contrasts with prior studies linking ACEs to hypertension through mental health pathways, but aligns with emerging evidence suggesting depression’s effects may be: Sample specificity of depression’s effects [e.g., Somatization in older adults may mask true depression levels (42)] and Subgroup differences in depression manifestation (43); Measurement limitations (Short-term assessments may fail to capture chronic effects (44) and Current scales inadequately assess chronicity (45)).

The results of this study reveal that, among individuals aged 45 and older in China, at least 50.23% reported experiencing at least one ACE, a finding consistent with existing literature (34, 46). Previous studies have highlighted the high prevalence of ACEs across diverse populations, with figures as high as 91.7% reported among incarcerated individuals (47). This study revealed a higher prevalence of physical abuse (28.3%) compared to other ACE types. While direct cross-study comparisons are limited by methodological differences, this finding aligns with three key evidence-based patterns from the literature: First, regarding population-level ACE burden, research on Korean college students (*n* = 939) found that 50% reported at least one ACE category, confirming high baseline exposure across cultures. Physical abuse accounted for 22.7% of these ACEs, with 8% of subjects experiencing four or more ACE categories (48). Second, in terms of the differential impact of abuse types, physical abuse exhibits distinct mechanistic pathways to adverse health outcomes: It has a direct effect on childhood depression without requiring emotional comorbidities (30, 31). It shows stronger predictive validity for depression than emotional abuse (43). Finally, the observed consistency may reflect contextual risk amplification: Socioeconomic stress potentiation: Low-income groups exhibit 1.8 × higher ACE exposure, with physical abuse mediating poverty-depression pathways (25). Behavioral transmission cycles: Childhood physical abuse predicts adult intimate partner violence victimization (OR = 2.15), contributing to intergenerational continuity.

Consistent with previous literature, this study confirms that ACEs are positively associated with hypertension, suggesting that individuals who endure traumatic childhood experiences, such as neglect, abuse, or household dysfunction, are at an increased risk of developing high blood pressure later in life (49, 50). However, our findings also suggest that ACEs do not directly cause hypertension; rather, their influence is mediated through cardiometabolic dysregulations.

The relationship between ACEs and hypertension has been widely investigated in both medical and psychological literature. Our study contributes to this body of work by demonstrating that depressive symptoms mediate the effect of ACEs on hypertension. This aligns with previous research showing a strong correlation between ACEs and the onset of depressive symptoms (51, 52). ACEs can influence the development of depressive symptoms through various psychosocial mechanisms. Individuals who have experienced childhood trauma often internalize feelings of worthlessness and helplessness, which heighten their vulnerability to stress and emotional distress in adulthood (53, 54). Additionally, the absence of a stable emotional support system during childhood can impair coping abilities, further increasing the risk of depression (55). Emotional dysregulation, a frequent outcome of childhood trauma, has also been closely linked to ACEs, with affected individuals often experiencing difficulty managing emotions, which predisposes them to depressive symptoms (56).

Our findings also reveal a significant association between depressive symptoms and hypertension, corroborating previous studies that highlight this relationship across diverse populations (57, 58). This evidence suggests that depression may serve as a key risk factor for the development of hypertension. Prolonged psychological stress and depressive symptoms can disrupt the function of the hypothalamic–pituitary–adrenal (HPA) axis, which plays a critical role in regulating blood pressure (55). In individuals with hypertension, depressive symptoms are closely linked to the activation of the HPA axis, which may contribute to fluctuations and increases in blood pressure (59). Additionally, depression may influence hypertension through its effects on the endocrine system. Dysregulation of the endocrine system associated with depressive states can lead to an overactive sympathetic nervous system, which further exacerbates increases in blood pressure (20).

Additionally, our findings suggest that ACEs may contribute to the development of hypertension through the pathway of cardiometabolic disorders. We observed a significant positive correlation between ACEs and cardiometabolic dysfunction, which aligns with previous research in the field (60, 61). ACEs can trigger stress responses at both physiological and psychological levels, leading to conditions such as chronic inflammation, hormonal imbalances, and autonomic nervous system dysregulation. These factors have been identified as potential contributors to the onset of cardiometabolic disorders (61, 62).

ACEs are increasingly recognized as significant risk factors for cardiovascular health. In the present study, we found a robust association between cardiometabolic dysfunction and hypertension. Emerging research indicates that cardiometabolic disorders, particularly those related to insulin resistance, lipid metabolism abnormalities, and inflammatory responses, play a pivotal role in the onset and progression of hypertension (63–65). This underscores the importance of addressing metabolic health in individuals with a history of ACEs, suggesting that interventions aimed at improving metabolic function could serve as a critical strategy in mitigating hypertension risk within this vulnerable population.

Our follow-up analysis revealed the emergence of 434 new hypertension cases, with males accounting for 53.2% of the total. This finding underscores the necessity for further exploration of gender differences in the relationship between ACEs and hypertension. Prior research has suggested that significant gender-related differences exist in psychological and physiological responses, which may subsequently influence the incidence and manifestation of hypertension (66).

Future research should aim to elucidate the causal relationships among ACEs, depressive symptoms, cardiometabolic dysregulation, and hypertension, while also considering the impact of gender differences on these interactions. Understanding the interplay of these factors is essential, as it would facilitate the identification of high-risk populations and provide valuable insights into the development of effective prevention and intervention strategies. By addressing these complex relationships, we can work towards reducing the incidence of hypertension and its associated complications, ultimately contributing to improved public health outcomes across diverse demographic groups.

This study has several limitations that must be acknowledged. First, the categories of ACEs included in our research are not comprehensive, as they do not account for experiences of sexual abuse. Second, the retrospective nature of ACE reporting may introduce recall bias and social desirability bias, potentially affecting the validity of our findings. Third, inconsistencies in the categorization of ACEs across studies complicate direct comparisons with prior research. Fourth, the two mediating variables—depressive symptoms and cardiometabolic dysregulation—were treated as parallel mediators, which may influence the results and their interpretation. Fifth, A key limitation of this study is the absence of data on Positive Childhood Experiences (PCEs). While current evidence does not directly validate PCEs as ACEs buffers, the literature demonstrates that: Self-compassion independently weakens depression pathways for emotional abuse victims, yet its interaction with ACEs was unmeasured (30, 31); Methodological homogeneity persists across ACEs research—all retrieved studies exclusively used risk-focused measures (e.g., 70.8% depression in ≥4 ACEs groups) without resilience assessment. Finally, while hypertension is widely recognized as being associated with unhealthy lifestyle choices, our study did not incorporate lifestyle factors as mediators, which may limit the understanding of the full scope of the relationship.

Based on the findings, intervention strategies could be considered from the following perspectives. First, establish a linkage between ACEs screening and hypertension risk alert systems, incorporating psychological assessments into routine health checks. Second, develop integrated intervention programs combining psychological counseling and metabolic management for high-risk groups. Third, promote trauma-informed care models within primary healthcare settings. Implementing these measures would require policy support, including the integration of ACEs assessment into public health monitoring, training healthcare professionals to identify trauma-related health risks, and developing multidisciplinary clinical guidelines. Future research should focus on evaluating the cost-effectiveness and implementation pathways of such interventions (48). Future research should address this gap by embedding validated cognitive-affective measures (30, 31) and tracking biological trajectories longitudinally (45).

In conclusion, prospective data from this study indicate that approximately half of the population experiences ACEs, and a positive correlation exists between ACEs and an increased incidence of hypertension. Notably, ACEs do not appear to exert a direct effect on hypertension; rather, this relationship is mediated by depressive symptoms and cardiometabolic dysregulation. Future studies should address the limitations of this research by expanding the categories of ACEs assessed, employing longitudinal designs to minimize recall bias, and incorporating lifestyle factors into the analysis. Such efforts would deepen our understanding of the pathways linking ACEs to hypertension and ultimately inform the development of more effective prevention and intervention strategies.

## Data Availability

Publicly available datasets were analyzed in this study. This data can be found at: https://charls.charlsdata.com/index/zh-cn.html.
